# Visual mapping of body image disturbance in anorexia nervosa reveals objective markers of illness severity

**DOI:** 10.1038/s41598-021-90739-w

**Published:** 2021-06-10

**Authors:** Christina Ralph-Nearman, Armen C. Arevian, Scott Moseman, Megan Sinik, Sheridan Chappelle, Jamie D. Feusner, Sahib S. Khalsa

**Affiliations:** 1 Laureate Institute for Brain Research (LIBR), Tulsa, OK USA; 2 Department of Psychological and Brain Sciences, University of Louisville, Louisville, KY USA; 3 Jane and Terry Semel Institute for Neuroscience and Human Behavior, University of California Los Angeles, Los Angeles, CA USA; 4 Laureate Psychiatric Clinic and Hospital, Tulsa, OK USA; 5 Oxley College of Health Sciences, University of Tulsa, Tulsa, OK USA; 6 Centre for Addiction and Mental Health, Toronto, ON Canada; 7 Department of Psychiatry, University of Toronto, Toronto, ON Canada

**Keywords:** Psychology, Biomarkers, Health care, Medical research, Risk factors, Signs and symptoms

## Abstract

Body image disturbance (BID) is a core feature of eating disorders, for which there are few objective markers. We examined the feasibility of a novel digital tool, “Somatomap”, to index BID related to anorexia nervosa (AN) severity. Fifty-five AN inpatients and 55 healthy comparisons (HC) outlined their body concerns on a 2-Dimensional avatar. Next, they indicated sizes/shapes of body parts for their current and ideal body using sliders on a 3-Dimensional avatar. Physical measurements of corresponding body parts, in cm, were collected for reference. We evaluated regional differences in BID using proportional z-scores to generate statistical body maps, and multivariate analysis of covariance to assess perceptual discrepancies for current body, ideal body, and body dissatisfaction. The AN group demonstrated greater regional perceptual inaccuracy for their current body than HC, greater discrepancies between their current and ideal body, and higher body dissatisfaction than HCs. AN body concerns localized disproportionately to the chest and lower abdomen. The number of body concerns and perceptual inaccuracy for individual body parts was strongly associated with Eating Disorder Examination Questionnaire (Global EDE-Q) scores across both groups. Somatomap demonstrated feasibility to capture multidimensional aspects of BID. Several implicit measures were significantly associated with illness severity, suggesting potential utility for identifying objective BID markers.

## Introduction

Body image disturbance (BID), defined as disruption of how one’s body size is experienced, is a core diagnostic feature for anorexia nervosa (AN)^1^, which is among the most deadly of all psychiatric illnesses^1,2^. BID is associated with a higher risk of relapse^3,4^, which occurs in nearly half of all patients^5^, suggesting that a greater insight into the perceptual mechanisms underlying this process may be key for more effective and durable treatments. BID is a multifaceted construct that can be loosely divided into perceptual^6–9^ and attitudinal^10–12^ components. As there is a subjective element of this construct, it is difficult to assess the more objective sensory and affective components of how an individual perceives their own body, and few assessment tools exist to capture this.

Clinical measures of BID (e.g., the Body Image States Scale)^10^, commonly require patients to select from a predetermined menu of word indicators to describe their mentalized representation of their body. Perceptual inaccuracy (i.e., discrepancies between the person’s receipt of body signals and their corresponding interpretation) has been suggested to be a core characteristic of AN for decades^13–15^, and has been demonstrated empirically in multiple studies^6^. These perceptual distortions lead to both distress and subsequent eating disorder behaviors. For instance, an individual with AN may view their overall body or certain parts of their body as extremely large, when it is actually very thin. These perceptual inaccuracies and distortions are often related to negative emotions. Eating disorder behaviors may partly reflect attempts to correct misperceptions of body size and shape, and to temporarily reduce negative emotions, which are ultimately ineffective. Therefore, assessing the degree of these perceptual distortions as an indicator of eating disorder illness severity may inform treatment targets. Yet, it is still unclear whether and to what degree these abnormalities are due to a primary dysfunction of sensory encoding or another perceptual misrepresentation mechanism. Moreover, BID in AN has been linked to disrupted activity and connectivity in visual processing and parietal association networks^16–20^, raising the possibility that these brain areas play a disproportionate role in developing and/or maintaining over-estimations of one’s body size and shape.

Visual silhouette assessments^21–24^ are one commonly used method to overcome reliance on language-based inferences. Some figure scales have strong psychometric properties, provide quick assessments of overall body dissatisfaction^24^, and measure distinct dimensions of body dissatisfaction independently (e.g., adiposity/muscularity)^21,22^. Yet, they do not measure other details such as the types of body concerns (e.g., fat, acne, sweat), lack a focus on specific body parts (e.g. abdominal protrusion, thighs, neck, etc.), and they do not specifically measure the associated emotional impact of body concerns. Visual computer-based body-shaping tools have been developed, but they also typically only assess overall body size perception because they use multiple body parts that scale together, or else use preset virtual avatars^25–29^. This precludes identification of which aspects of the body are key to generating the disturbance, making it impossible to pinpoint the precise body areas that are misperceived as problematic, distorted, or obese, and to link these misperceptions with negative affect and eating disorder behaviors. Moreover, most tools also obscure the head/neck, preventing the identification of BID related to these features. The available studies assessing distinct body part distortions have found that individuals with AN overestimate individual body parts more than the whole body size^6,30^, suggesting a need for visual tools with this capability.

We recently created a multi-platform digital tool called “Somatomap”, which utilizes a visual representation of specific body concern areas on 2-dimensional (2D) and 3-dimensional (3D) avatars to facilitate BID assessment related to specific regions across the entire body. In a proof-of-concept study with female professional fashion models and nonmodels, this tool identified patterns of body image concerns localized to discrete body areas (see Ralph-Nearman et al. 2019^31^ for development details). These results raised the possibility that Somatomap might have utility for investigation of BID in clinical populations, such as in individuals with eating disorders.

The current study examined the feasibility of measuring BID characteristics in individuals with AN using Somatomap. In transitioning Somatomap for use from a nonclinical (female fashion models^31^) to a clinical sample we made several refinements to the original tool, including increasing the number of 3D body part measurements to 23 independently manipulatable body areas and assessing additional body image measures (ideal body image perception). This allowed us to estimate a measure of “body dissatisfaction”, defined by subtracting the ideal from the currently perceived body rating. We also evaluated the relationship between Somatomap BID measures and traditional estimates of illness severity (i.e., EDE-Q). We predicted that inpatients with AN (relative to healthy comparisons, HC) would exhibit greater BID on the visual measures in Somatomap as evidenced by a significantly higher proportion of body image concern areas (Somatomap 2D), and significantly lower perceptual accuracy for the size/shape of individual body parts (Somatomap 3D). We also predicted that the degree of BID on Somatomap 2D and 3D would be associated with eating disorder symptom severity as measured by the EDE-Q. Finally, we employed a usability scale with the prediction that both groups would report acceptable levels of usability for each measure.

## Methods

### Participants

We recruited individuals diagnosed with AN from the Laureate Eating Disorders Inpatient Program in Tulsa, Oklahoma, and HCs from the Tulsa community. Clinical inclusion criteria included a primary diagnosis of AN by the treating psychiatrist as noted in their medical records during admission, body mass index (BMI) $\ge$ 14, female sex (a requirement for treatment), age from 13 to 64 years, and independent ambulation. Exclusion criteria included active suicidal ideation or a comorbid psychotic disorder. HCs were recruited from the local community, screened for the presence of DSM-5 diagnoses via the MINI structured clinical interview^32^, and met the eligibility criteria for age, sex, and independent ambulation.

### BID measurement via Somatomap

#### Somatomap 2D

Participants first used a mouse to outline an area of body concern directly on a 2D human avatar presented on a computer screen. They subsequently selected the specific concerns (e.g., “too fat”, “acne”, etc.) and the emotions they related to each area (e.g., “fearful”, “disgusted”, “other [be specific]”, etc.) from a menu of visual icons. In addition to the 39 specific types of body concerns and the 26 specific emotions icon word-pairs they could select from, a free text-entry option was available for participants to indicate any other specific body concern areas or any other specific emotions related to their body concerns not listed in the menu.

Participants also rated the level of defectiveness, emotion intensity, and perceived distress surrounding their body concern using visual-analogue scales (from 0 = not at all to 100 = extremely). This process was repeated for each individual body concern. There was no limit on the number of body concerns that they could outline and rate, and no time limit.

#### Somatomap 3D

After personalizing the avatar to match their own hair and skin color, the 3D avatar was displayed on screen. The size/shape of the 23 adjustable body parts were randomly permuted for the initial avatar to prevent potential priming effects associated with viewing anatomically proportioned bodies^33^. Participants used sliders to adjust the shape of each 3D avatar body area independently, in order to show how they perceived their current body, and then again to show how they would like their ideal body to look. Adjustable body areas included: neck girth, neck length, shoulder width, bust girth, chest girth, biceps girth, upper arms length, forearms girth, lower arms length, wrists girth, hands girth, hands length, torso length, waist, stomach form, hips, thighs girth, thighs length, calves girth, calves length, ankles, feet width, and feet length.

The protocol for obtaining the physical body measurements of participants was applied from our prior Somatomap study^31^ (adapted from the PhenX toolbox^34^), with the inclusion of 16 additional body areas for a total of 23 body areas. Areas with multiple parts (e.g., hands, feet, thighs, biceps, forearms, etc.) were measured individually and then averaged. We used the InBody bioimpedance scale (Cerritos, California) and a stadiometer to calculate each participant’s actual body mass index (BMI).

### Eating disorder symptom assessment

To estimate the degree of eating disorder symptom severity, each participant completed the Eating Disorder Examination Questionnaire (EDE-Q 6.0)^35^, which includes four subscales: Shape, Weight, Eating, and Restraint concern. A summed average of the four subscale ratings yields an EDE-Q Global score; all scores range across a scale from 0-none to 6-severe. Scores ≥ 4.0 have often been considered to reflect clinically severe levels of eating disorder psychopathology^36,37^.

### Data collection procedure

This study was approved by the Western Institutional Review Board, and all methods were carried out in accordance with relevant guidelines and regulations. Prior to the experiment, each participant provided written informed consent, and informed consent was obtained from a parent and/or legal guardian for the participants under 18 years of age (Trial Registration: ClinicalTrials.gov #NCT03758326). All participants completed demographic questions, BID assessments (Somatomap 2D and 3D in counterbalanced order), and self-report scales at LIBR on a computer. User experience surveys were collected after completing the 2D and 3D measures, and physical measurements were completed last. The data for the Somatomap app and survey was collected on the Chorus app platform^38^. Chorus is a visual development platform supporting the creation of web-based digital health applications that can be accessed on various devices including mobile, tablet and desktop.

### Statistical analysis

#### Somatomap 2D

To create group-level proportional maps of body concerns, all pixelwise body concern tracings were merged for each person (overlapping pixels were set to 1) and the ensuing binary maps were overlapped as in our prior Somatomap study procedure^31^. To evaluate between-group differences in the body maps we used custom Matlab software (Mathworks, Inc.) to calculate the test statistic for each pixel using the z-formula for proportion, and employed cluster correction (6 pixel threshold) and smoothing following our previously described procedures^31,39,40^. Across both groups, a linear regression model (lm) conducted in R (version 3.6.2) examined the relationship between the number of individual body concerns traced and eating disorder symptomatology (EDE-Q Global Scores).

#### Somatomap 3D

The 3D assessment procedure yielded six distinct scores across each of the 23 body parts: (1) actual body, (2) current perceived body, (3) ideal perceived body, (4) current body discrepancy (2 − 1), (5) ideal body discrepancy (3 − 1), and (6) body dissatisfaction (3 − 2). Both current and ideal perceived body measurement scores were converted into centimeters from arbitrary units using piecewise linear interpolation as previously described^31^. Similar to our previous study, we used multivariate analysis of covariance (MANCOVA) to determine if there were group differences in each of these measurements. Covariates included BMI, height, and weight. If the MANCOVA results were significant, a follow-up analysis using analysis of covariance (ANCOVA) with the Benjamini–Hochberg multiple comparison correction was used to determine which variables showed statistically significant differences between ANs and HCs. Multiple linear regression models were used to examine relationships between body dissatisfaction body part measures and body discrepancy body part measures (separately) with measures of psychopathology severity (EDE-Q), across both groups. Multiple linear regression models were used to examine whether intersecting measures on 2D and 3D together might explain more variance of symptom severity than when applied with 2D concerns and 3D body dissatisfaction and body discrepancy separately.

#### User experience

Usability surveys were collected after completing both measures to assess the ease and enjoyability of use, the degree to which the 2D tool effectively captured participants’ BID concerns and associated emotions, and the degree of identification with the original and final 3D avatar.

## Results

### Participant demographics

Fifty-five AN and 55 HC females completed testing (see Table 1 for key demographic data; Fig. S.1. for Consort diagram). The AN group had a significantly lower BMI than HC (*P* < 0.001), which was driven by differences in body weight (*P* < 0.001) but not height (*P* = 0.70). No significant differences for overall race/ethnicity (*χ*^2^_4_ = 7.36; *P* = 0.061), or education levels (*χ*^*2*^_5_ = 10.90; *P* = 0.053) between the groups were identified.

**Table 1:** Demographic and clinical characteristics of anorexia nervosa inpatients and healthy comparisons.

	AN, mean (SD)	HC, mean (SD)	t (*df*)	*P-*value
Gender	55 females	55 females		
Age (years)	25.25 (11.00)	23.42 (4.98)	1.13 (75)	0.26
Height (cm)	163.92 (6.60)	164.47 (8.40)	− 0.38 (102)	0.70
Weight (kg)	50.67 (7.75)	61.13 (7.27)	− 7.30 (108)	< 0.001
Body mass index (kg/m^2^)	18.85 (2.83)	22.61 (2.25)	− 7.70 (103)	< 0.001
**Race/ethnicity (n, %)**			*χ*^2^_4_ = 7.36	0.061
White/Caucasian	50 (90.90)	40 (72.73)		
Black	0 (0)	2 (3.63)		
Asian (including East Indian)	0 (0)	2 (3.63)		
More than one race/ethnicity^a^	5 (9.10)	11 (20.00)		
**Education**			*χ*^2^_5_ = 10.90	0.053
Graduate school	7 (12.73)	8 (14.55)		
University graduate	9 (16.36)	19 (34.55)		
Some university	22 (40.00)	23 (41.82)		
High school/A level/GED	11 (20.00)	3 (5.45)		
Some high school/A level	4 (7.27)	2 (3.64)		
Less than high school/A level	2 (3.64)	0 (0)		
EDE-Q Global	4.25 (1.09)	0.65 (0.66)	20.95 (108)	< 0.001
Age of onset (years)	14.7 (3.1)	–		
Illness duration (years)	10.2 (11.4)	–		
Psychotropic medication (%)	89.1	–		
**Comorbid diagnoses (n, %)**^**b**^				
Major depressive disorder	24 (42.6)	–		
Generalized anxiety disorder	21 (38.2)	–		
OCD	17 (30.9)	–		
PTSD	9 (16.4)	–		

*GED* general educational development, *OCD* obsessive compulsive disorder, *PTSD* posttraumatic stress disorder.

^a^Hispanic/Latino descent, Lebanese, American Indian/Alaska Native, Native Hawaiian or other Pacific Islander, Jewish, Black, White/Caucasian, Asian (including East Indian) listings per the PhenX^34^.

^b^For brevity, only comorbid diagnoses with > 10% frequency are listed.

### Somatomap 2D

**Figure 1:**
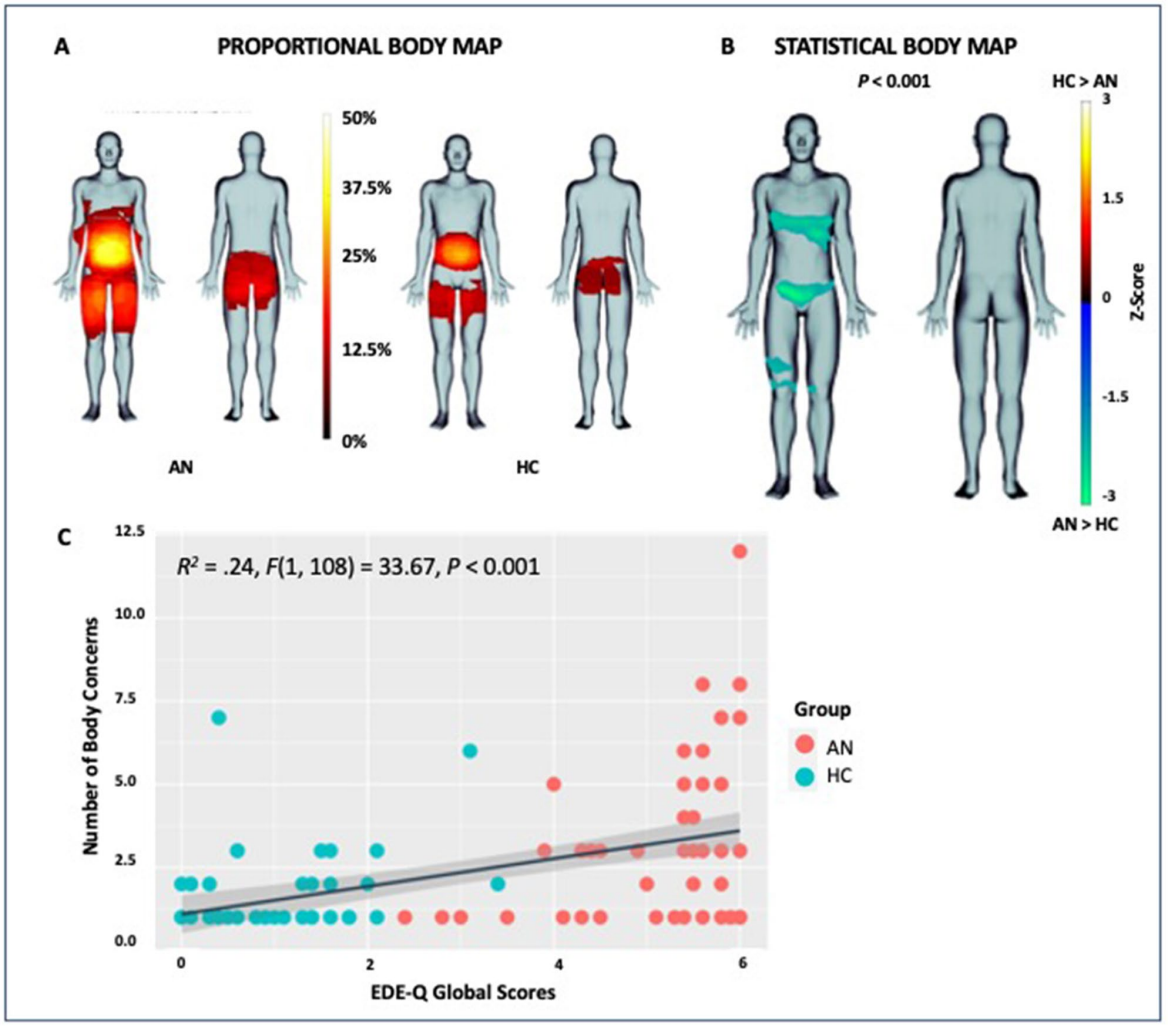
(**A**) (top left) Proportional body maps displaying the majority of body concern areas for each group (left anorexia nervosa (AN) group; middle healthy comparisons (HC)). (**B**) (top right) Statistical body map evaluating differences in body image concerns between AN group (in cool colors) and HCs (in warm colors; statistical threshold = *P* < 0.001); (**C**) (bottom) Association between Number of Body Concerns and Eating Disorder Symptomatology (EDE-Q Global Scores) across ANs and HCs (*P* < 0.001).

Proportional body maps showed that AN participants perceived body concerns across a broader area compared with HCs (Fig. 1A). The statistical body map analysis revealed that the AN group perceived significantly more concerns localized to the lower abdomen and chest than HCs (*P* < 0.001) (Fig. 1B). The number of body concerns outlined ranged from 1 to 12 per individual for ANs (*M* = 3.20; *SD* = 2.45) and from 1 to 7 per individual for HCs (*M* = 1.55; *SD* = 1.17), which significantly differed between groups (*t*_108_ = 4.52, *P* < 0.001). Across both groups, a linear regression revealed a significant relationship between the Global EDE-Q score and the number of body concern areas outlined (*R*^2^ = 0.24, *F* = 33.67, *P* < 0.001; Fig. 1C). EDE-Q Global concerns were reliable (Cronbach’s $\alpha$=0.98). Participants took an average of 2.2 min (*SD* = 1.6) to complete Somatomap 2D (AN *M* = 2.6, *SD* = 1.8; HC *M* = 1.8, *SD* = 1.3).

The number of body concern types identified (e.g., “too fat”, “bloated”) ranged from 1 to 73 per individual for ANs (*M* = 15.65*; SD* = 15.74) and from 0 to 20 per individual for HCs (*M* = 3.53; *SD* = 4.11), which was significantly different between groups (*t*_108_ = 1.98, *P* < 0.001). The number of affective labels selected (e.g., “frustrated”, “angry”, “disgusted”, etc.) for all concerns ranged from 0 to 93 per individual for ANs (*M* = 16.3; *SD* = 18.2) and from 1 to 22 per individual for HCs (*M* = 2.6; *SD* = 3.9); which was also significantly different between groups (*t*_109_ = 5.4, *P* < 0.001; Table S.1).

Emotion ratings associated with body concerns were significantly higher for ANs relative to HCs (*Ps* < 0.001) with regards to the level of defectiveness (AN (*M* = 60.70; *SD* = 23.24); HC (*M* = 21.34; *SD* = 16.26)), emotion intensity (AN (*M* = 56.70; *SD* = 26.05); HC (*M* = 23.35; *SD* = 27.73)), and degree of distress caused by body concerns (AN (*M* = 63.25; *SD* = 25.61); HC (*M* = 12.33; *SD* = 14.42)).

### Somatomap 3D

#### Actual body, current perceived body, and ideal perceived body

There were multiple statistically significant group differences in the measurements of the actual body, current perceived body, and ideal perceived body. A complete listing of these results is provided in the Supplement. Here, we summarize the statistically significant differences observed. The AN group had a significantly smaller measured bust girth, chest, biceps, waist, “stomach form” (abdominal protrusion), thighs, and calves, and had a shorter upper arm length compared with HCs (MANCOVA: *F*(23, 83) = 33.73, Wilks Λ = 0.097, *P* < 0.001; Table S.2). However, they perceived various current body girths to be significantly larger than the HC group (i.e., chest, biceps, waist, stomach form, thighs, calves, neck, forearms, hips, and ankles; MANCOVA: *F*(23, 83) = 1.73, Wilks Λ = 0.675, *P* = 0.037; Table S.3). The AN and HC groups showed few differences in idealized body girth and length characteristics, except that the AN group desired narrower shoulders, a longer torso, smaller hips, calves, and feet width than the HC group (MANCOVA: *F*(23, 83) = 3.40, Wilks Λ = 0.515, *P* < 0.001; Table S.4).

#### Current body discrepancy, ideal body discrepancy, and body dissatisfaction

**Figure 2:**
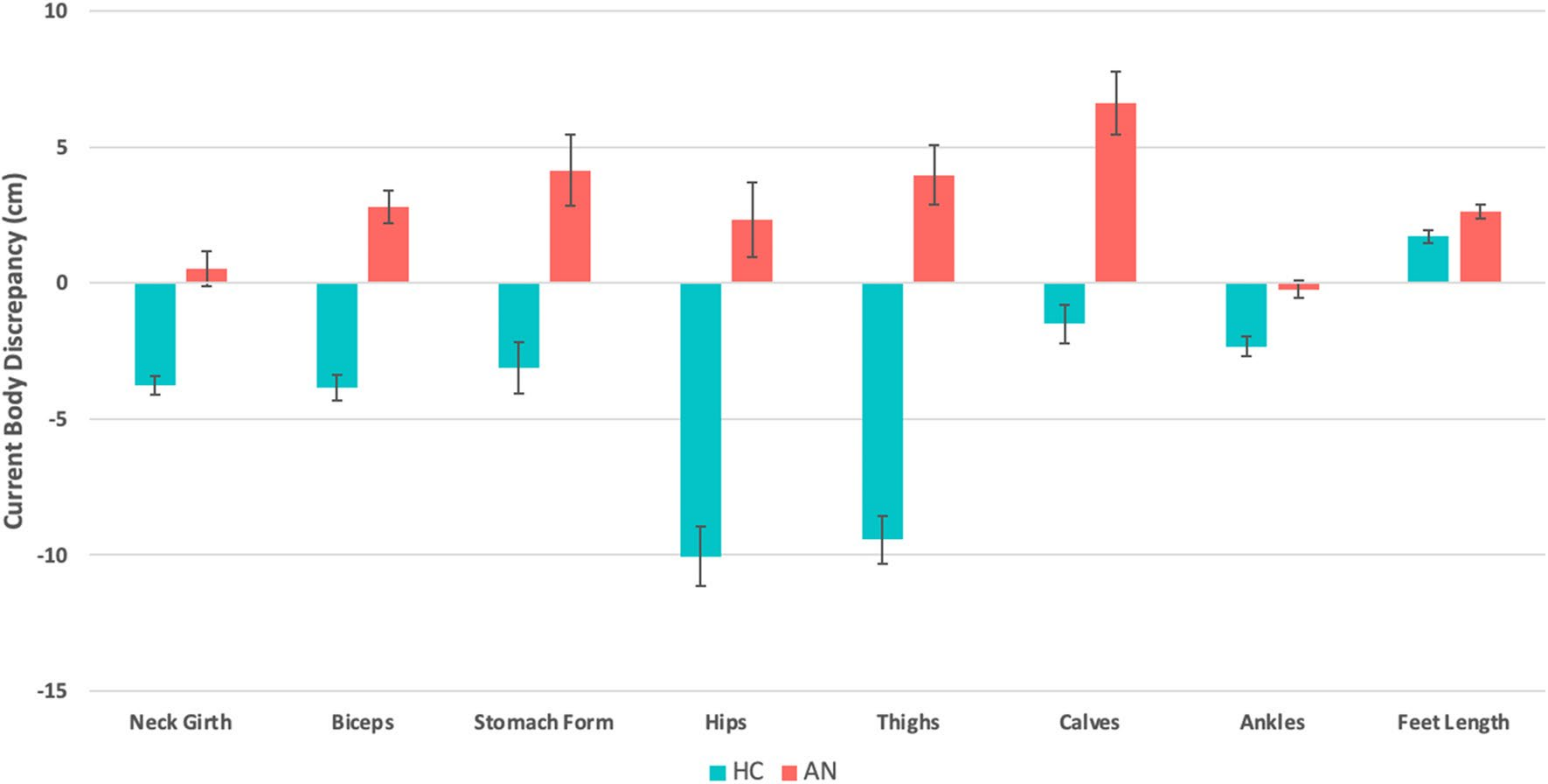
Summary of current body discrepancy score differences between AN and HC. Of the total set of 23 body part measurements only those showing significant differences are displayed (P < 0.05, corrected for multiple comparisons using the Benjamini–Hochberg procedure). Positive values indicate overestimation of true body part size, negative values indicate underestimation, and zero indicates correct estimation. Standard errors are represented by error bars.

There were multiple statistically significant differences in the current body discrepancy, ideal body discrepancy, and body dissatisfaction measurements. A complete listing of these results is provided in the Supplement. In summary, the AN group had significantly higher discrepancy scores (overestimated current body sizes) for neck girth, biceps, stomach form, hips, thighs, calves, ankles, feet length, and overall body compared to HCs (MANCOVA: *F*(23, 83) = 11.89, Wilks Λ = 0.232, *P* < 0.001; Fig. 2, Table S.5).

**Figure 3:**
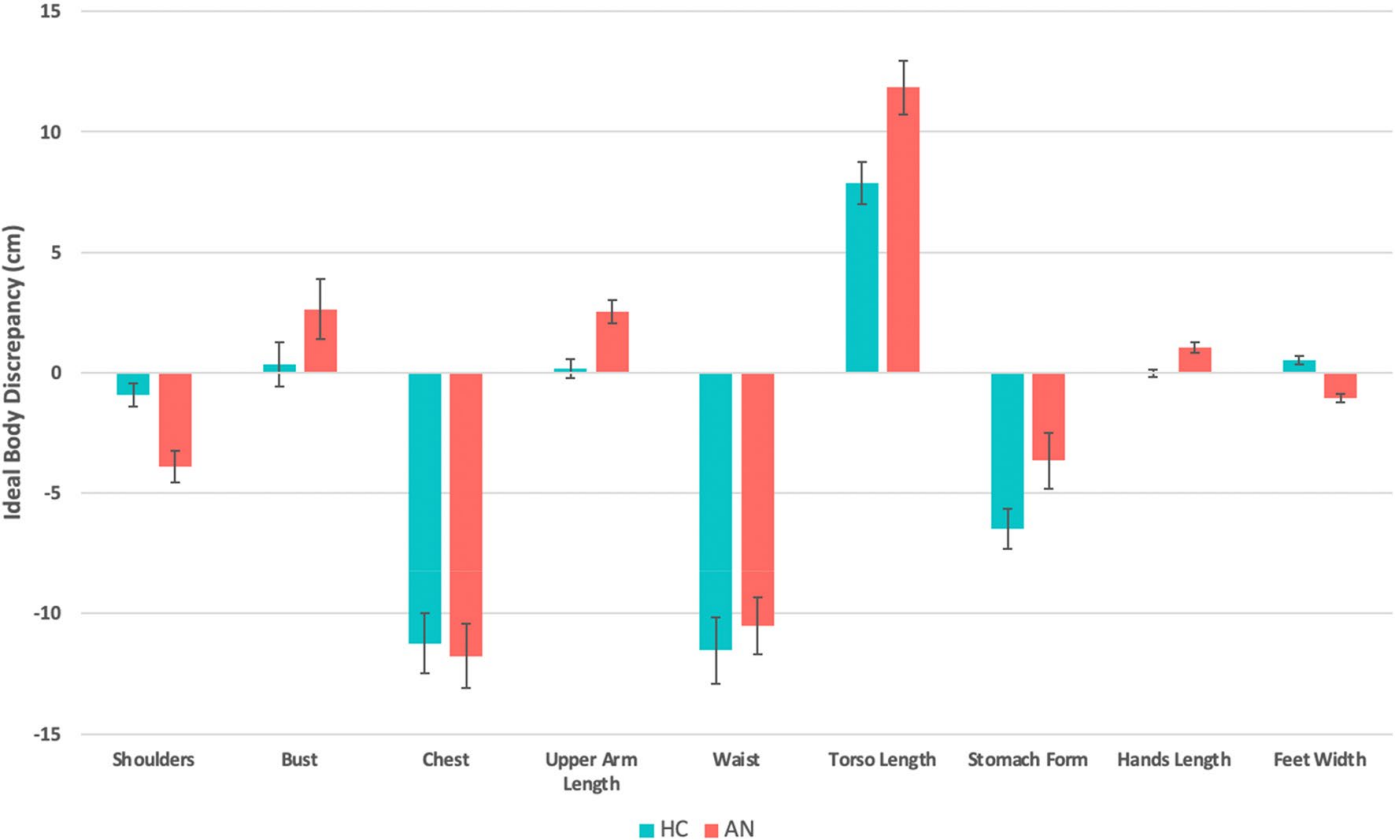
Ideal body discrepancy (ideal body minus actual body measurements (cm)) in female AN and HC groups. Of the total set of 23 body part measurements only those showing significant differences are displayed (*P* < 0.05, corrected for multiple comparisons using the Benjamini–Hochberg procedure). Positive values indicate ideal body part size is larger than true body part size, negative values indicate ideal body part size is smaller than true body part size, and zero indicates the ideal and true body part size is the same. Standard errors are represented by error bars.

Relative to HCs, the AN group had significant differences in ideal body discrepancy scores such that they desired narrower shoulders, a thinner chest, a larger bust, a thinner waist, narrower feet, longer upper arms, torsos, and hands, compared with their actual body part sizes and shapes. Both groups desired a thinner stomach form, but the AN group desired it to be significantly thinner relative to their actual body (MANCOVA: *F*(23, 83) = 6.37, Wilks Λ = 0.362, *P* < 0.001; Fig. 3, Table S.6).

**Figure 4:**
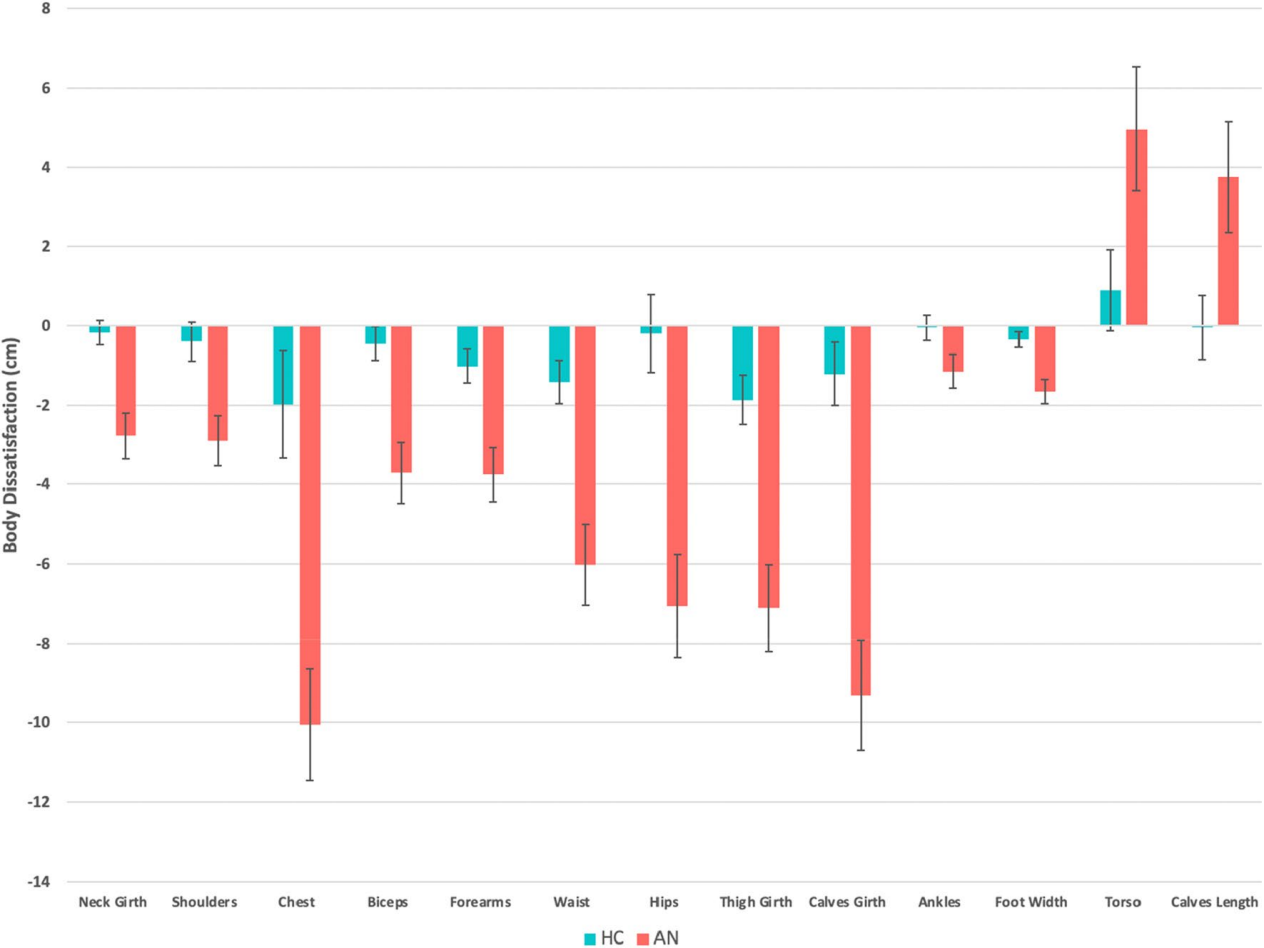
Body Dissatisfaction Score (ideal minus current perceived (cm)) in female AN and HC groups. All body parts refer to girth except where indicated for length. Of the total set of 23 body part measurements only those showing significant differences are displayed (*P* < 0.05, corrected for multiple comparisons using the Benjamini–Hochberg procedure). Standard errors are represented by error bars.

The AN group showed significantly greater body dissatisfaction scores for neck girth, chest, biceps, forearms, waist, hips, thighs, calves, ankles, feet width, and overall body (towards the thin ideal) relative to the HC group (MANCOVA: *F*(23, 83) = 2.59, Wilks Λ = 0.582, *P* < 0.001; Fig. 4, Table S.7). They also had greater dissatisfaction for their shoulders (preferring them narrower), torso and calves (preferring them longer) (see Table 2 for a summary of significant group differences for each of the six measures). Somatomap 3D took participants 6.6 min on average to complete (AN *M* = 6.9; *SD* = 2.2; HC *M* = 6.4; *SD* = 2.0).

**Table 2:** Summary of statistically significant AN vs. HC body part differences across all six Somatomap 3D measures.

Measure	Girth	*Cohen’s f*	Length	*Cohen’s f*
Actual body	*AN narrower than HC*		*AN shorter than HC*	
	Bust	*0.37*	Upper arms	*0.387*
	Chest	*0.350*		
	Biceps	*0.318*		
	Waist	*0.288*		
	Stomach form	*0.405*		
	Thighs	*0.367*		
	Calves	*0.299*		
Current perceived	*AN overestimated*		No differences	
	Neck	0.246		
	Chest	0.351		
	Biceps	0.358		
	Forearms	0.275		
	Waist	0.359		
	Stomach form	0.238		
	Hips	0.282		
	Thighs	0.296		
	Calves	0.318		
	Ankles	0.350		
Current body discrepancy	*AN overestimated*		*AN overestimated*	
	Neck	*0.289*	Feet length	*0.157*
	Biceps	*0.481*		
	Stomach form	*0.447*		
	Hips	*0.308*		
	Thighs	*0.495*		
	Calves	*0.403*		
	*AN underestimated*			
	Ankles	*0.264*		
Ideal perceived	*AN desired smaller*		*AN desired longer*	
	Shoulders	*0.311*	Torso	*0.243*
	Hips	*0.269*		
	Calves	*0.245*		
	Feet width	*0.258*		
Ideal body discrepancy	*AN desired smaller*		*AN desired longer*	
	Shoulders	*0.316*	Torso	*0.307*
	Chest	*0.240*	Upper arms	*0.306*
	Waist	*0.335*	Hands	*0.238*
	Stomach form	*0.302*		
	Feet width	*0.252*		
	*AN desired larger*			
	Bust	*0.374*		
Body dissatisfaction	*AN desired smaller*		*AN desired longer*	
	Neck	*0.252*	Torso	*0.257*
	Shoulders	*0.238*	Calves	*0.251*
	Chest	*0.348*		
	Biceps	*0.345*		
	Forearms	*0.243*		
	Waist	*0.401*		
	Hips	*0.420*		
	Thighs	*0.351*		
	Calves	*0.426*		
	Ankles	*0.273*		
	Feet width	*0.296*		

23 body part assessments (15 girth/8 length) were examined for each measure.

#### Regression analysis

##### 3D body dissatisfaction and ED severity

We conducted a multiple linear regression using the EDE-Q Global as the dependent variable, with body dissatisfaction scores on 3D for all body parts as independent variables, across groups. Body dissatisfaction parts on 3D were significantly associated with participants’ eating disorder psychopathology severity on the EDE-Q (*R*^2^ = 0.50, *F*(23,86) = 3.71, *P* < 0.001) (see Fig. S.2).

##### 3D current body discrepancy and ED severity

Next, we conducted a multiple linear regression to investigate the relationships between EDE-Q scores and the current body discrepancy scores on 3D for all body parts, across groups. Body discrepancy on 3D was significantly associated with eating disorder symptom severity on the EDE-Q, accounting for 67% of the variance of global psychopathology (*R*^*2*^ = 0.67; *F*(23,86) = 7.57, *P* < 0.001) (see Fig. S.3).

#### Aggregated 2D with 3D body dissatisfaction and ED severity

To explore the degree to which aggregating the number of body concerns on 2D and all of the body dissatisfaction parts on 3D, were related to eating disorder severity, we conducted a multiple linear regression using the EDE-Q Global scores as the dependent variable, with the number of 2D body concerns plus all of the body dissatisfaction parts on 3D as independent variables, across groups. Together, the number of 2D concerns with all of the 3D body dissatisfaction body parts explained 59% of the variance of overall ED psychopathology (*R*^2^ = 0.59, *F*(24,85) = 5.03, *P* < 0.001).

##### 3D chest and waist body dissatisfaction with number of 2D concerns and ED severity

Chest and waist were body areas identified by both the 2D and 3D approaches as being significantly related to BID in AN relative to HCs. We conducted a multiple linear regression between the number of 2D body concerns and 3D chest and waist body dissatisfaction and the EDE-Q Global scores, which explained 40% of the variance of overall ED psychopathology (*R*^2^ = 0.40, *F*(3,106) = 23.4, *P* < 0.001).

##### Aggregated 3D current body discrepancy with number of 2D concerns and ED severity

Finally, to examine the degree to which aggregating relevant 2D and 3D measures explained symptom severity, we conducted a multiple linear regression using the EDE-Q Global as the dependent variable, with the number of 2D body concerns plus all of the current body discrepancy parts on 3D as independent variables, across groups. Together, the number of 2D concerns with all of the 3D current body discrepancy body parts explained a high percentage of the variance of overall ED psychopathology (*R*^2^ =0.73; *F*(24,85) = 9.73, *P* < 0.001). All pre-checks for all regression models performed showed minimal concern evidence for of multicollinearity (variance inflation factor < 5).

##### Usability assessment

Participants rated the 2D body map and 3D avatar as easy to use, as very reflective of their body concerns (2D), and that the final 3D avatar was mostly reflective of their actual perceived body (Tables S.8, S.9). HCs reported significantly higher levels of ease (2D: *t*_91_ = 2.48, *P* = 0.015; 3D: *t*_108_ = 2.69, *P* = 0.008), and enjoyment (2D: *t*_91_ = 4.04, *P* = 0.001; 3D: *t*_108_ = 3.69, *P* < 0.001), and how closely the final 3D avatar reflected their body (*t*_108_ = 2.92, *P* < 0.004) than the AN group. Participants “liked least” that Somatomap: “was lacking muscle tone”, “started randomized”, and “liked best” that it was: “easy to use”, “enabled me to express myself”, and “very realistic”.

## Discussion

The current study tested the feasibility of a novel digital tool for assessing multidimensional BID characteristics, including objective measurements of perceptual disturbance for body part size estimation and subjective experiences of dissatisfaction, in acutely ill individuals with AN relative to HCs. We also tested the relationships between these measures and illness severity estimates. We found, as hypothesized, that there were significant BID differences between groups in terms of perceptual overestimations, thin-ideal body dissatisfaction, and body concerns (for individual body part size/shape on Somatomap 3D as well as the total number of body concerns on Somatomap 2D), and in terms of the affective valence associated with body concerns. Finally, Somatomap 2D and 3D difference measures were associated with symptom severity measures across groups when examined individually and when aggregrated, with aggregated measures explaining the greatest symptom variance.

Analysis of the Somatomap 2D data revealed that this tool detected a greater degree of BID concerns in AN than HCs for the chest and abdomen, with greater levels of perceived distress, intensity, and defectiveness related to AN body concerns. Moreover, across both groups, the number of outlined body concerns was positively associated with eating disorder symptom severity on the EDE-Q when controlling for age and BMI. This result raises the intriguing notion that a single perceptual rating about body concerns (involving a few mouse clicks and minutes) could provide information about an individual’s eating disorder psychopathology severity. Such an implicit approach could potentially augment or replace more time-consuming screening methods which explicitly signal the search for a disorder, although additional study is needed to verify this possibility. At a minimum, this approach provides a clear indication of the visual perceptual body mapping and associated emotional characteristics related to BID in AN, at the individual and group levels.

Analysis of the Somatomap 3D data identified that, overall, the AN group (relative to HCs) showed a general over-estimation of current body girth and a preference for a thinner body than what they currently perceived. This was apparent from the current perceived body, current body discrepancy, ideal perceived body discrepancy, and body dissatisfaction scores, which were associated with symptom severity. These findings support the value of assessments of individual body parts to better understand perceptual and attitudinal aspects of BID. They raise the possibility of impaired cognitive and/or visual mechanisms operating in individuals with AN^7,20,41^, which might decrease their ability to accurately perceive their own body.

Our observation that the AN group overestimated body sizes and reported increased negative emotion compared with HCs supports the self-discrepancy theory^42^, which suggests that mismatches between actual and ideal internal self-representations produce negative affect and poor health outcomes. These results also support a recent meta-analysis which found that more negative and less positive affect ranges for self-discrepancy were significantly related to psychopathology^43^. We might speculate that the AN inpatients’ overestimations of their body size compared to HCs in our current study indicates an inability or difficulty to calibrate their body perception in relation to the general population. Another possibility is that they may ‘re-calibrate’ their body perception towards other underweight individuals present in their current treatment setting. The development and/or maintenance of body size and shape over-estimations in AN may be related to previously demonstrated brain activation and connectivity abnormalities in visual systems. For example, BID in AN may also be due to a disturbance in brain connectivity and abnormal functioning in body processing networks^44,45^. This might involve reduced connectivity between the left fusiform body area and extrastriate body area^44–46^, reduced neural activity in cortical visual systems and hyperconnectivity in dorsal visual and parietal networks^20^, which may be associated with body size misperception^44–46^ in AN. The connectivity of these networks could be further investigated in future studies utilizing Somatomap. For a discussion of the relevance of these findings to theories of body perception in HCs, see the Supplement.

Two truncal areas of AN body concern were identified across Somatomap 2D and 3D: the abdomen and chest. Excessive fullness and/or bloating of the stomach and intestines is a frequent complaint for individuals with AN, which are often identified in clinical settings as motivators of restrained eating^25^. The chest is another focus area given that it is a region containing adipose tissue in females. Both regions show a lower percentage of body fat in the underweight stage followed by an increased ratio of truncal/extremity fat during weight restoration^26^, which may help to explain the common overlap across measures. Our exploratory aggregated regression analyses revealed that these measures explained a substantial degree of symptom severity on the EDE-Q. Specifically, while the chest and waist body dissatisfaction (3D) together with the number of 2D body concerns explained 40% of the variance of EDE-Q, and body dissatisfaction parts (3D) together with the number of 2D body concerns explained 59% of the EDE-Q variance, it was the 3D current discrepancy body parts (together with the number of 2D body concerns) that explained the most variance of symptom severity: 73% of the EDE-Q Global score variance. Although exploratory, these results suggest that combining convergent measures focusing on the visual perceptual characteristics or body dissatisfaction of discrete body parts, and total number of body parts of concern, might be used to make meaningful inferences about the degree of symptom severity across the spectrum of eating psychopathology.

Objectively distinguishing the perceptual and affective components of BID may be critical to better understanding AN, in terms of screening, prevention, diagnostic assessment, prognostic prediction, intervention targeting, and relapse prevention monitoring. Clinicians currently measure BID by relying upon subjective, language-based questionnaires or visual analogue scales which assess body dissatisfaction and body distortion constructs in a global manner. The present study tested the feasibility of a different approach, one that included quantification of visual perceptual distortions and body dissatisfaction for discrete body parts. This revealed: (1) both Somatomap 2D and 3D detected visual perceptual and affective aspects of BID in AN, explained unique variance in eating disorder symptom severity and greater variance when combined across groups, (2) both were tolerable, with no one quitting the measurement during test/retest, (3) usability was generally good, although the significantly reduced usability rating in AN (compared with HCs) means there is room for participant-suggested improvements, and (4) Use of a multi-platform digital tool means it can be remotely deployed such as in post-acute settings for monitoring and detecting illness trajectory. In addition to providing more specific information about distinct combinations of BID and illness severity, this tool may equip researchers to visually, statistically, and remotely pinpoint BID parameters that are uniquely related to ED severity.

### Limitations, strengths and future directions

This study has several limitations. First, AN participation in this study was limited to females from a single inpatient treatment facility, and therefore, testing with a broader range of facilities, care settings, and demographic characteristics (including males) would be important to demonstrate generalizability. Second, although enjoyability of the tool was rated by AN participants above average, this experience could be further improved (particularly in the AN patient group) by incorporating AN participants’ usability assessment feedback to include other dimensions of the body (e.g., they suggested adding muscularity). Longitudinal and reliability studies will be necessary to test the tool’s ability to detect changes in illness severity (e.g., relapse/remission/recovery), and comparing this assessment with other measures of BID to highlight the incremental validity will be essential. An additional approach could evaluate how changes in BID relate to (or predict) longitudinal clinical outcomes. A strength of the current approach is that it effectively captures a broad range of body dissatisfaction self-concepts in relation individual body parts, and illustrates their association with illness severity. The digital multi-platform nature of this tool could have broad applications including remote deployment, in multiple clinical settings.

### Conclusion

The current study demonstrates the initial feasibility of Somatomap for assessing multidimensional aspects of BID related to ED severity in AN. The novel visual perceptual mapping approach entailed by this tool can capture implicit responses and estimate perceptual disturbances at the level of individual body parts, yielding objective markers of BID. This positions Somatomap as a potentially useful tool both for research and clinical applications.

## Supplementary Information

Supplementary Information.
