# Supplementary material for: Visual mapping of body image disturbance in anorexia nervosa reveals objective markers of illness severity
Source: Sci Rep. 2021 Jun 10;11:12262. doi: 10.1038/s41598-021-90739-w (PMC8192536; doi:10.1038/s41598-021-90739-w)
Supplement: Supplementary file 1 — Supplementary Information. [file 41598_2021_90739_MOESM1_ESM.docx]

**Visual mapping of body image disturbance in anorexia nervosa reveals objective markers of illness severity**

**Christina Ralph-Nearman, MS, PhD^1,2 *^, Armen C. Arevian, MD, PhD^3^,**

**Scott Moseman, MD^4^, Megan Sinik^1^, Sheridan Chappelle^1^,**

**Jamie D. Feusner, MD^3^, and Sahib S. Khalsa, MD, PhD^1,5^**

**^1^Laureate Institute for Brain Research (LIBR), Tulsa, Oklahoma**

**^2^Department of Psychological and Brain Sciences, University of Louisville, Kentucky**

**^3^Jane and Terry Semel Institute for Neuroscience and Human Behavior, University of California Los Angeles**

**^4^Laureate Psychiatric Clinic and Hospital, Tulsa, Oklahoma**

**^5^Oxley College of Health Sciences, University of Tulsa, Tulsa, Oklahoma**

**Running head:** VISUAL MAPPING OF BODY IMAGE DISTURBANCE

***Corresponding authors:**

Dr. Christina Ralph-Nearman

University of Louisville

Department of Psychological & Brain Sciences

Louisville, Kentucky

United States

Email: ChristinaRalphNearman@gmail.com

Tel: +1 424-634-0920

Dr. Sahib Khalsa

Laureate Institute for Brain Research

Tulsa, Oklahoma

United States

Email: skhalsa@laureateinstitute.org

**Supplement**

*Supplemental Discussion:*

In contrast to the AN group’s body size overestimations, the observed HC underestimations of body size aligns with results reported in a prior study with women in the general population.^30^ These findings support the self-serving bias theory that healthy individuals tend to underestimate their body size, which may preserve a more positive body attitude relative to AN.^46^ It may be that HCs’ visual body perception adapts in correspondence to the body size exposure (the population around them), as visual attention toward body types with high or low adiposity manipulates body misperception in women with healthy BMI, distinctly from body dissatisfaction.^47^ Presently, as the HC’s BMI was on average in the lower, ‘healthy’ range (*M*=22.61), and much slimmer than 66.9% of women in the general United States population,^48^ it may be that these individuals calibrated to the larger general population, underestimating their own body size. In fact, the social norm hypothesis proposes that healthy, overweight, or obese individuals calibrate the perception of their own body size perception compared to the body size of the growing general population.^49^

**Supplementary Tables**

**Table S.1**. Frequency and percentage of participants endorsing emotions (at least once) related to each area body concern.

| Emotion type | AN  *n* (%) | HC  *n* (%) |
| --- | --- | --- |
| **Negative** | | |
| Frustrated | 40 (72.7) | 19 (34.6) |
| Anxious, tense, worried, nervous | 51 (92.7) | 4 (7.3) |
| Disgusted | 48 (87.3) | 6 (10.9) |
| Ashamed | 45 (81.8) | 5 (9.1) |
| Sad | 31 (56.4) | 6 (10.9) |
| Depressed | 36 (65.5) | 2 (3.6) |
| Hopeless | 20 (36.4) | 2 (3.6) |
| Overwhelmed | 28 (50.9) | 1 (1.8) |
| Fearful | 21 (38.2) | 1 (1.8) |
| Angry | 15 (27.3) | 0 (0) |
| Rejected | 11 (20.0) | 0 (0) |
| Lonely | 7 (12.7) | 0 (0) |
| Numb/unreal/dead | 7 (12.7) | 0 (0) |
| Other (e.g., grossed out, annoyed, guilty, displeased, nasty, apathetic, self-conscious, insecure etc.) | 2 (3.6) | 3 (5.5) |
| Embarrassed | 2 (3.6) | 0 (0) |
| **Neutral/positive** | | |
| Looks ok/fine | 2 (3.6) | 33 (60.0) |
| Hopeful | 3 (5.5) | 10 (18.2) |
| Satisfied/content | 1 (1.8) | 3 (5.5) |
| Relieved | 1 (1.8) | 0 (0) |

**Table S.2.** Actual body measurements in centimeters for the AN and HC groups. Statistically significant findings in bold.

**Actual body MANCOVA: F^b^(23, 83) = 33.73, Wilks Λ^b^ = 0.097, *P^b^* < 0.001**

| *Variable* | *HC, mean (SD)* | *AN, mean (SD)* | *P value^a^* | *Partial* η^2^ | *Cohen’s f* |
| --- | --- | --- | --- | --- | --- |
| Neck Girth | 30.80 (2.41) | 28.64 (3.21) | 0.31 | .016 | 0.128 |
| Neck Length | 13.91 (1.44) | 13.64 (1.60) | 0.79 | .0009 | 0.031 |
| Shoulders | 31.69$($2.96) | 33.36 (4.33) | 0.14 | .031 | 0.178 |
| **Bust Girth** | **87.00**$\mathbf{(}$**5.01)** | **83.45**$\mathbf{(}$**6.60)** | **0.002** | **.123** | **0.374** |
| **Chest Girth** | **85.87 (5.85)** | **81.55 (6.04)** | **0.003** | **.109** | **0.350** |
| **Biceps Girth** | **27.02**$\mathbf{(}$**3.04)** | **22.25**$\mathbf{(}$**2.95)** | **0.008** | **.092** | **0.318** |
| **Upper Arms Length** | **33.35 (2.41)** | **31.09 (2.26)** | **0.001** | **.130** | **0.387** |
| Forearms Girth | 19.98 (3.35) | 18.09 (1.78) | 0.97 | .00002 | 0.005 |
| Lower Arms Length | 25.60 (1.68) | 25.11 (1.79) | 0.65 | .004 | 0.060 |
| Wrists | 14.55 (0.81) | 14.15 (1.33) | 0.36 | .013 | 0.113 |
| **Waist** | **71.67(9.78)** | **69.51**$\mathbf{(}$**8.33)** | **0.015** | **.076** | **0.288** |
| Torso Length | 44.42 (3.49) | 43.55 (4.50) | 0.29 | .017 | 0.132 |
| Hands Girth | 17.91 (0.99) | 17.35 (1.17) | 0.55 | .006 | 0.077 |
| Hands Length | 17.40 (1.05) | 16.96 (0.96) | 0.44 | .010 | 0.098 |
| **Stomach Form** | **38.16 (4.69)** | **33.82 (5.65)** | **<0.001** | **.114** | **0.405** |
| Hips | 99.46$($6.71) | 88.93$($7.76) | 0.41 | .011 | 0.103 |
| **Thighs Girth** | **56.49**$\mathbf{(}$**5.50)** | **46.29**$\mathbf{(}$**6.79)** | **0.002** | **.118** | **0.367** |
| Thighs Length | 48.78 (3.90) | 49.62 (4.54) | 0.72 | .002 | 0.045 |
| **Calves Girth** | **35.31**$\mathbf{(}$**2.28)** | **31.13**$\mathbf{(}$**3.28)** | **0.011** | **.082** | **0.299** |
| Calves Length | 42.45 (3.64) | 40.49 (3.66) | 0.41 | .011 | 0.107 |
| Ankles | 21.2 (1.47) | 20.36 (2.24) | 0.70 | .002 | 0.049 |
| Feet Width | 8.44 (0.79) | 8.60 (0.68) | 0.89 | .0002 | 0.015 |
| Feet Length | 22.8 (1.28) | 22.53 (1.43) | 0.44 | .001 | 0.037 |
| **Scaled Body Average** | **0.987**$\mathbf{(}$**0.04)** | **0.986**$\mathbf{(}$**0.05)** | **<0.001** | **.488** | **0.958** |

^a^*P* values corrected for multiple comparisons using the Benjamini-Hochberg procedure.

^b^Measured using multivariate analysis of covariance.

**Table S.3.** Current perceived body measurements in centimeters for the AN and HC groups. Statistically significant findings in bold.

**Current Perceived Body MANCOVA: F^b^(23, 83) = 1.73, Wilks Λ^b^ = 0.675, *P^b^* = 0.037**

| *Variable* | *HC, mean (SD)* | *AN, mean (SD)* | *P-value^a^* | *Partial* η^2^ | *Cohen’s f* |
| --- | --- | --- | --- | --- | --- |
| **Neck Girth** | **26.96 (1.66)** | **29.15 (3.75)** | **0.037** | **.057** | **0.246** |
| Neck Length | 15.80$($3.14) | 16.35$($3.30) | 0.67 | .003 | 0.054 |
| Shoulders | 31.13$($2.99) | 32.40$($4.31) | 0.44 | .009 | 0.097 |
| Bust Girth | 85.18$($5.08) | 85.75$($6.01) | 0.30 | .017 | 0.130 |
| **Chest Girth** | **76.58**$\mathbf{(}$**8.30)** | **79.85**$\mathbf{(}$**7.07)** | **0.003** | **.110** | **0.351** |
| **Biceps Girth** | **23.16**$\mathbf{(}$**2.41)** | **25.05**$\mathbf{(}$**4.03)** | **0.003** | **.113** | **0.358** |
| Upper Arms Length | 33.44 (3.02) | 32.89 (3.54) | 0.62 | .004 | 0.063 |
| **Forearms Girth** | **14.49 (2.43)** | **16.15 (3.60)** | **0.02** | **.070** | **0.275** |
| Lower Arms Length | 22.07 (4.13) | 22.29 (4.63) | 0.77 | .001 | 0.037 |
| Wrists | 11.75 (1.60) | 12.47 (2.16) | 0.06 | .047 | 0.222 |
| **Waist** | **61.60 (4.64)** | **64.98 (6.14)** | **0.003** | **.114** | **0.359** |
| Torso Length | 51.44 (6.69) | 50.53 (9.18) | 0.47 | .008 | 0.089 |
| Hands Girth | 17.58 (1.05) | 17.73 (1.50) | 0.31 | .016 | 0.127 |
| Hands Length | 17.47 (1.14) | 17.75 (1.40) | 0.98 | .00003 | 0.005 |
| **Stomach Form** | **35.06 (5.74)** | **37.96 (8.34)** | **0.042** | **.054** | **0.238** |
| **Hips** | **89.36 (6.97)** | **91.24 (9.00)** | **0.016** | **.074** | **0.282** |
| **Thighs Girth** | **47.05 (4.55)** | **50.27 (6.33)** | **0.012** | **.081** | **0.296** |
| Thighs Length | 54.62 (4.79) | 56.00 (6.22) | 0.55 | .006 | 0.076 |
| **Calves Girth** | **33.8 (5.13)** | **37.75 (8.44)** | **0.008** | **.092** | **0.318** |
| Calves Length | 43.69 (5.74) | 41.58 (8.00) | 0.06 | .047 | 0.221 |
| **Ankles** | **18.87 (2.00)** | **20.13 (2.10)** | **0.003** | **.109** | **0.350** |
| Feet Width | 8.56 (1.21) | 9.20 (1.74) | 0.18 | .026 | 0.163 |
| Feet Length | 24.53 (1.93) | 25.15 (2.11) | 0.11 | .035 | 0.191 |
| Scaled Body Average | 0.98 (0.05) | 0.98 (0.07) | 0.68 | .002 | 0.044 |

^a^*P* values corrected for multiple comparisons using the Benjamini-Hochberg procedure.

^b^Measured using multivariate analysis of covariance.

**Table S.4.** Ideal body measurements in centimeters for the AN and HC groups. Statistically significant findings in bold.

**Ideal Perceived Body MANCOVA: F^b^(23, 83) = 3.40, Wilks Λ^b^ = 0.515, *P^b^* < 0.001.**

| *Variable* | *HC, mean (SD)* | *AN, mean (SD)* | | *P-value^a^* | *Partial* η^2^ | *Cohen’s f* |
| --- | --- | --- | --- | --- | --- | --- |
| Neck Girth | 26.76(1.77) | | 26.25(2.00) | 0.40 | .012 | .108 |
| Neck Length | 16.53(3.18) | | 17.20(3.11) | 0.27 | .019 | .138 |
| **Shoulders** | **30.78(2.77)** | | **29.40(1.98)** | **0.009** | **.088** | **.311** |
| Bust Girth | 87.33(5.14) | | 86.04(5.75) | 0.10 | .037 | .197 |
| Chest Girth | 74.65(7.77) | | 69.95(7.67) | 0.36 | .013 | .116 |
| Biceps Girth | 22.62(2.38) | | 21.35(3.08) | 0.27 | .019 | .138 |
| Upper Arms Length | 33.51(2.86) | | 33.64(3.20) | 0.44 | .010 | .098 |
| Forearms Girth | 13.51(2.16) | | 12.35(2.98) | 0.47 | .008 | .089 |
| Lower Arms Length | 23.20(3.34) | | 22.36(4.33) | 0.38 | .012 | .109 |
| Wrists | 11.36(1.53) | | 10.89(1.88) | 0.91 | .0001 | .012 |
| Waist | 60.13(3.31) | | 58.96(3.64) | 0.26 | .020 | .144 |
| **Torso Length** | **52.29(6.34)** | | **55.38(7.99)** | **0.039** | **.056** | **.243** |
| Hands Girth | 17.04(0.88) | | 16.95(1.13) | 0.98 | .000008 | .003 |
| Hands Length | 17.38(1.03) | | 18.02(1.16) | 0.069 | .044 | .215 |
| Stomach Form | 31.67(5.00) | | 30.16(5.56) | 0.89 | .0004 | .021 |
| **Hips** | **89.20(5.75)** | | **84.11(4.76)** | **0.023** | **.067** | **.269** |
| Thighs Girth | 45.18(2.89) | | 43.13(4.51) | 0.13 | .032 | .181 |
| Thighs Length | 56.38(5.21) | | 58.02(5.38) | 0.27 | .019 | .141 |
| **Calves Girth** | **32.58(5.37)** | | **28.44(5.86)** | **0.038** | **.057** | **.245** |
| Calves Length | 43.67(5.73) | | 45.35(7.44) | 0.44 | .009 | .096 |
| Ankles | 18.69(1.65) | | 18.84(2.23) | 0.91 | .0002 | .014 |
| **Feet Width** | **8.22(1.18)** | | **7.55(1.33)** | **0.03** | **.062** | **.258** |
| Feet Length | 24.71(1.52) | | 24.96(1.51) | 0.47 | .008 | .089 |
| Scaled Body Average | 0.98 (0.05) | | 0.98 (0.05) | 0.60 | .004 | .062 |

^a^*P* values corrected for multiple comparisons using the Benjamini-Hochberg procedure.

^b^Measured using multivariate analysis of covariance.

**Table S.5.** Current body discrepancy scores (perceived current minus actual body measurements) in centimeters for the AN and HC groups. Statistically significant findings in bold.

| **Current body discrepancy MANCOVA: F^b^(23, 83) = 11.89, Wilks Λ^b^ = 0.232, *P^b^* < 0.001** | | | | | | |  |
| --- | --- | --- | --- | --- | --- | --- | --- |
| *Variable* | *HC, mean (SD)* | *AN, mean (SD)* | *P-value^a^* | *Partial* η^2^ | | *Cohen’s f* | |
| **Neck Girth** | **–3.78 (2.58)** | **+0.53 (4.64)** | **0.015** | **.077** | **0.289** | | |
| Neck Length | +1.89$($3.23) | +2.71 (3.35) | 0.62 | .004 | 0.064 | | |
| Shoulders | –0.56 (3.83) | -0.93$($5.68) | 0.65 | .003 | 0.057 | | |
| Bust Girth | –1.80 (6.76) | +2.29 (7.10) | 0.42 | .001 | 0.100 | | |
| Chest Girth | –9.29$($8.25) | -1.64$($9.50) | 0.14 | .031 | 0.180 | | |
| **Biceps Girth** | **–3.85 (3.65)** | **+2.80 (4.57)** | **<.001** | **.188** | **0.481** | | |
| Upper Arms Length | +0.09$($2.87) | +1.80$($3.22) | 0.18 | .027 | 0.167 | | |
| Forearms Girth | -5.49 (3.92) | -1.95 (4.03) | 0.07 | .044 | 0.216 | | |
| Lower Arms Length | -3.53 (4.28) | -2.82 (5.09) | 0.70 | .003 | 0.051 | | |
| Wrists | -2.80 (1.67) | -1.67 (2.24) | 0.23 | .022 | 0.151 | | |
| Waist | -10.05 (10.31) | -4.49 (9.09) | 0.77 | .001 | 0.036 | | |
| Torso Length | +7.02 (6.38) | +6.98 (9.87) | 0.89 | .0003 | 0.018 | | |
| Hands Girth | -0.33 (1.43) | +0.38 (1.78) | 0.23 | .022 | 0.151 | | |
| Hands Length | +0.07 (1.27) | +0.78 (1.54) | 0.66 | .003 | 0.055 | | |
| **Stomach Form** | **-3.11 (7.04)** | **+4.15 (9.74)** | **<.001** | **.166** | **0.447** | | |
| **Hips** | **-10.05 (7.97)** | **+2.33 (10.30)** | **0.009** | **.087** | **0.308** | | |
| **Thighs Girth** | **-9.44 (6.56)** | **+3.98 (8.18)** | **<.001** | **.197** | **0.495** | | |
| Thighs Length | +5.84 (4.83) | +6.38 (7.93) | 0.77 | .001 | 0.035 | | |
| **Calves Girth** | **-1.51 (5.17)** | **+6.62 (8.71)** | **<0.001** | **.140** | **0.403** | | |
| Calves Length | +1.24 (6.32) | +1.09 (8.38) | 0.23 | .023 | 0.152 | | |
| **Ankles** | **-2.33 (2.58)** | **-0.24 (2.36)** | **0.026** | **.065** | **0.264** | | |
| Feet Width | +0.18 (1.39) | +0.60 (1.80) | 0.27 | .019 | 0.140 | | |
| **Feet Length** | **+1.71 (1.82)** | **+2.62 (1.89)** | **0.036** | **.024** | **0.157** | | |
| **Scaled Body Average** | **–0.26**$\mathbf{(}$**0.29)** | **+0.19**$\mathbf{(}$**0.43)** | **.016** | **.066** | **0.266** | | |

^a^*P* values corrected for multiple comparisons using the Benjamini-Hochberg procedure.

^b^Measured using multivariate analysis of covariance.

**Table S.6.** Ideal body discrepancy scores (ideal minus actual body measurements) in centimeters for the AN and HC groups. Statistically significant findings in bold font.

| **Ideal body discrepancy MANCOVA: F^b^( 23, 83) = 6.37, Wilks Λ^b^ = 0.362, *P^b^* < 0.001.** | | | | | |
| --- | --- | --- | --- | --- | --- |
| *Variable* | *HC, mean (SD)* | *AN, mean (SD)* | *P-value^a^* | *Partial* η^2^ | *Cohen’s f* |
| Neck Girth | -3.96(3.25) | -2.31(3.35) | 0.75 | .002 | .039 |
| Neck Length | +2.62(3.22) | +3.56(3.19) | 0.26 | .020 | .142 |
| **Shoulders** | **-0.91(3.59)** | **-3.91(4.88)** | **0.008** | **.091** | **.316** |
| **Bust Girth** | **+0.33(6.82)** | **+2.64(9.27)** | **0.002** | **.123** | **.374** |
| **Chest Girth** | **-11.24(9.15)** | **-11.76(10.01)** | **0.04** | **.054** | **.240** |
| Biceps Girth | -4.40(3.80) | -0.91(4.16) | 0.42 | .010 | .101 |
| **Upper Arms Length** | **+0.16(2.89)** | **+2.55(3.57)** | **0.009** | **.086** | **.306** |
| Forearms Girth | -6.47(3.79) | -5.75(3.16) | 0.57 | .005 | .071 |
| Lower Arms Length | -2.40(3.42) | -2.75(4.51) | 0.32 | .015 | .124 |
| Wrists | -3.18(1.63) | -3.25(2.07) | 0.70 | .003 | .051 |
| **Waist** | **-11.53(10.22)** | **-10.51(8.61)** | **0.005** | **.101** | **.335** |
| **Torso Length** | **+7.87(6.39)** | **+11.84(8.32)** | **0.009** | **.086** | **.307** |
| Hands Girth | -0.87(1.14) | -0.40(1.68) | 0.68 | .003 | .054 |
| **Hands Length** | **-0.02(1.21)** | **+1.05(1.61)** | **0.042** | **.054** | **.238** |
| **Stomach Form** | **-6.49(6.26)** | **-3.66(8.45)** | **0.011** | **.083** | **.302** |
| Hips | -10.24(7.84) | -4.78(9.04) | 0.18 | .027 | .165 |
| Thighs Girth | -11.31(5.71) | -3.16(9.06) | 0.20 | .024 | .158 |
| Thighs Length | +7.60(5.32) | +8.40(7.18) | 0.49 | .007 | .085 |
| Calves Girth | -2.73(5.50) | -2.69(7.21) | 0.31 | .016 | .126 |
| Calves Length | +1.22(6.31) | +4.85(7.96) | 0.29 | .018 | .134 |
| Ankles | -2.51(1.70) | -1.53(3.27) | 0.72 | .002 | .045 |
| **Feet Width** | **+0.53(1.33)** | **-1.06(1.37)** | **0.033** | **.060** | **.252** |
| Feet Length | +1.89(1.58) | +2.44(1.74) | 0.27 | .016 | .040 |
| Scaled Body Average | –0.30$($0.30) | -0.13$($0.31) | 0.45 | .010 | .099 |

^a^*P* values corrected for multiple comparisons using the Benjamini-Hochberg procedure.

^b^Measured using multivariate analysis of covariance.

**Table S.7.** Body Dissatisfaction Score (ideal minus current perceived) in female anorexia nervosa and healthy comparison groups. Statistically significant findings in bold font.

| **Body dissatisfaction MANCOVA: F^b^( 23, 83) = 2.59, Wilks Λ^b^ = 0.582, *P^b^* < 0.001.** | | | | | |  |
| --- | --- | --- | --- | --- | --- | --- |
| *Variable* | *HC, mean (SD)* | *AN, mean (SD)* | *P-value^a^* | *Partial* η^2^ | *Cohen’s f* | |
| **Neck Girth** | **-0.18(2.25)** | **-2.78(4.26)** | **0.036** | **.060** | **.252** | |
| Neck Length | +0.71(2.91) | +0.85(3.00) | 0.44 | .009 | .096 | |
| **Shoulders** | **-0.40(3.65)** | **-2.91(4.71)** | **0.042** | **.054** | **.238** | |
| Bust Girth | +2.09(5.57) | +0.42(9.13) | 0.061 | .048 | .224 | |
| **Chest Girth** | **-1.98(10.03)** | **-10.05(10.38)** | **0.003** | **.108** | **.348** | |
| **Biceps Girth** | **-0.45(3.14)** | **-3.71(5.63)** | **0.003** | **.106** | **.345** | |
| Upper Arms Length | +0.02(2.17) | +0.71(3.72) | 0.26 | .020 | .144 | |
| **Forearms Girth** | **-1.02(3.10)** | **-3.75(5.00)** | **0.039** | **.056** | **.243** | |
| Lower Arms Length | +1.09(4.02) | +0.13(4.64) | 0.65 | .003 | .056 | |
| Wrists | -0.42(1.72) | -1.65(2.70) | 0.10 | .039 | .202 | |
| **Waist** | **-1.42(4.12)** | **-6.02(7.60)** | **<0.001** | **.139** | **.401** | |
| **Torso Length** | **+0.89(7.56)** | **+4.96(11.54)** | **0.03** | **.062** | **.257** | |
| Hands Girth | -0.55(1.17) | -0.76(1.70) | 0.36 | .013 | .116 | |
| Hands Length | -0.11(1.18) | +0.29(1.42) | 0.12 | .034 | .188 | |
| Stomach Form | -3.38(6.74) | -7.80(11.04) | 0.15 | .030 | .176 | |
| **Hips** | **-0.20(7.27)** | **-7.07(9.62)** | **<0.001** | **.150** | **.420** | |
| **Thighs Girth** | **-1.87(4.67)** | **-7.11(8.13)** | **0.003** | **.110** | **.351** | |
| Thighs Length | +1.80(4.27) | +1.93(6.50) | 0.71 | .002 | .048 | |
| **Calves Girth** | **-1.22(5.91)** | **-9.31 (10.28)** | **<0.001** | **.154** | **.426** | |
| **Calves Length** | **-0.04(5.98)** | **+3.75(10.31)** | **0.036** | **.059** | **.251** | |
| **Ankles** | **-0.05(2.37)** | **-1.15(3.13)** | **0.02** | **.070** | **.273** | |
| **Feet Width** | **-0.35(1.49)** | **-1.66(2.25)** | **0.012** | **.081** | **.296** | |
| Feet Length | +0.15(1.77) | -0.20(2.17) | 0.46 | .008 | .092 | |
| **Scaled Body Average** | **–0.07**$\mathbf{(}$**0.33)** | **-0.27**$\mathbf{(}$**0.40)** | **.019** | **.060** | **.254** | |

^a^*P* values corrected for multiple comparisons using the Benjamini-Hochberg procedure.

^b^Measured using multivariate analysis of covariance.

**Table S.8.** Somatomap 2D usability assessment results.

| Question | ANs  (*n*=38)^a^,  mean (*SD*) | HCs  (*n*=55),  mean (*SD*) |
| --- | --- | --- |
| 1. How easy was the 2D app to use? (1 - extremely difficult to 10 - extremely easy) Please explain. | 7.76 (2.54)^b^ | 8.85 (1.72)^b^ |
| 2. Was your experience using the 2D app? (1 - extremely frustrating to 10 - extremely enjoyable) Please explain. | 6.00 (2.04)^c^ | 7.75 (2.06)^c^ |
| 3. How well did the 2D app indicate your body concerns? (0 - Not at all to 10 - Completely) Please explain. | 7.79 (2.00) | 7.95 (2.35) |

^a^Only 38 of the 55 AN participants completed the 2D user experience questionnaire, because it was not available prior.

^b^*P*=0.015; ^c^*P*=0.001.

**Table S.9.** Somatomap 3D usability assessment results.

| Question | ANs  (*n*=55),  mean (*SD*) | HCs  (*n*=55),  mean (*SD*) |
| --- | --- | --- |
| 1. How easy was this app to use? (1 - Extremely difficult to 10 - Extremely easy) Please explain. | 6.71 (2.62)^a^ | 7.93 (2.10)^a^ |
| 2. What was your experience using this app? (1 - Extremely frustrating to 10 - Extremely enjoyable) Please explain. | 5.80 (2.37)^b^ | 7.42 (2.23)^b^ |
| 3. How much did you identify with the original 3D avatar? (0 - Not at all to 10 - Completely) Please explain. | 3.95 (3.44) | 3.45 (3.82) |
| 4. How closely did the final 3D avatar you created reflect your body? (0 - Not at all to 10 - Completely) Please explain. | 5.6 (3.5)^c,d^ | 7.2 (2.3)^c,d^ |

^a^*P*=0.008; ^b^*P*<0.001; ^c^*P*=0.004; ^d^This measure was collected on a 0 to 100 visual analogue scale, but the numbers have been converted to 0 to 10 scale to facilitate comparisons with other measures.

**Supplementary Figures**

**Figure S.1.** Consort Diagram


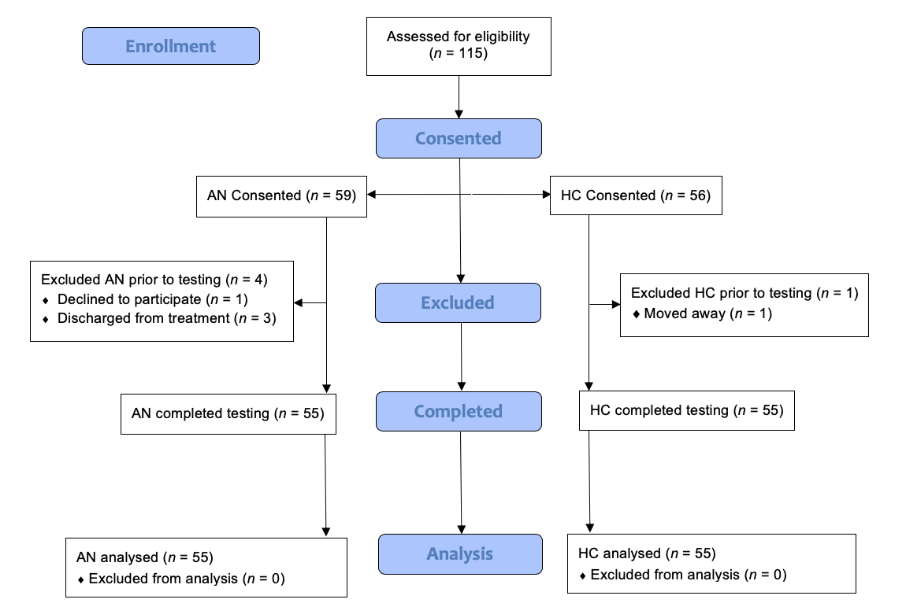


**Figure S.2.** 3D Body dissatisfaction parts (ideal perceived minus current perceived cms) were significantly associated with participants’ eating disorder symptom severity on the EDE-Q, across groups. 3D Body Dissatisfaction with negative values indicates ideal body is thinner than current perceived body part size, positive values indicate ideal body is larger than current perceived body part size, and zero indicates ideal body and current perceived body are similar. Body dissatisfied toward the thin-ideal was related to higher eating disorder severity, across groups.


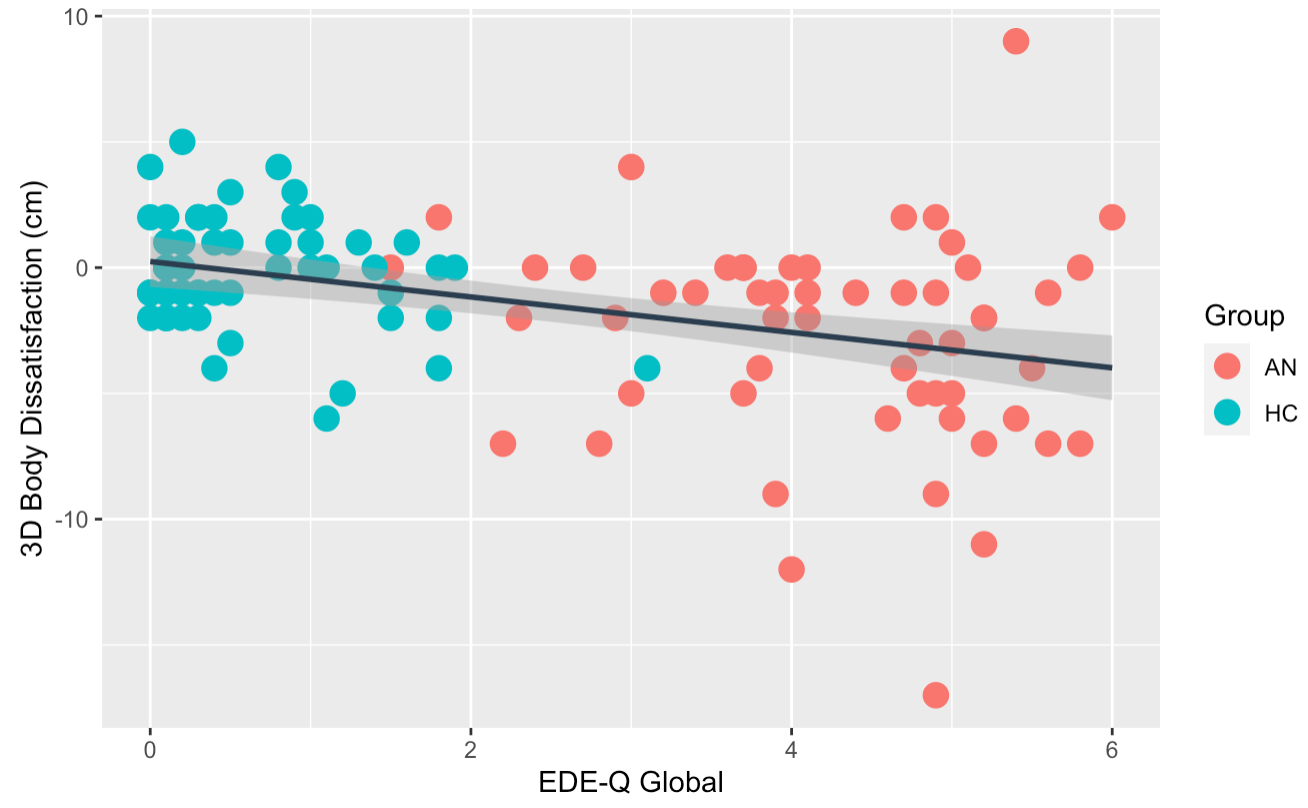


*R^2^* = .50, *F* (23,86) = 3.71, *P* < 0.001

**Figure S.3.** 3D Current Body discrepancy (perceived current minus actual body measurements) in centimeters were significantly associated with participants’ eating disorder symptom severity on the EDE-Q, across groups. 3D Current Body Discrepancies with positive values indicate overestimation of true body part size, negative values indicate underestimation, and zero indicates correct estimation. Over-estimation of body parts was related to increased eating disorder severity, across groups.*
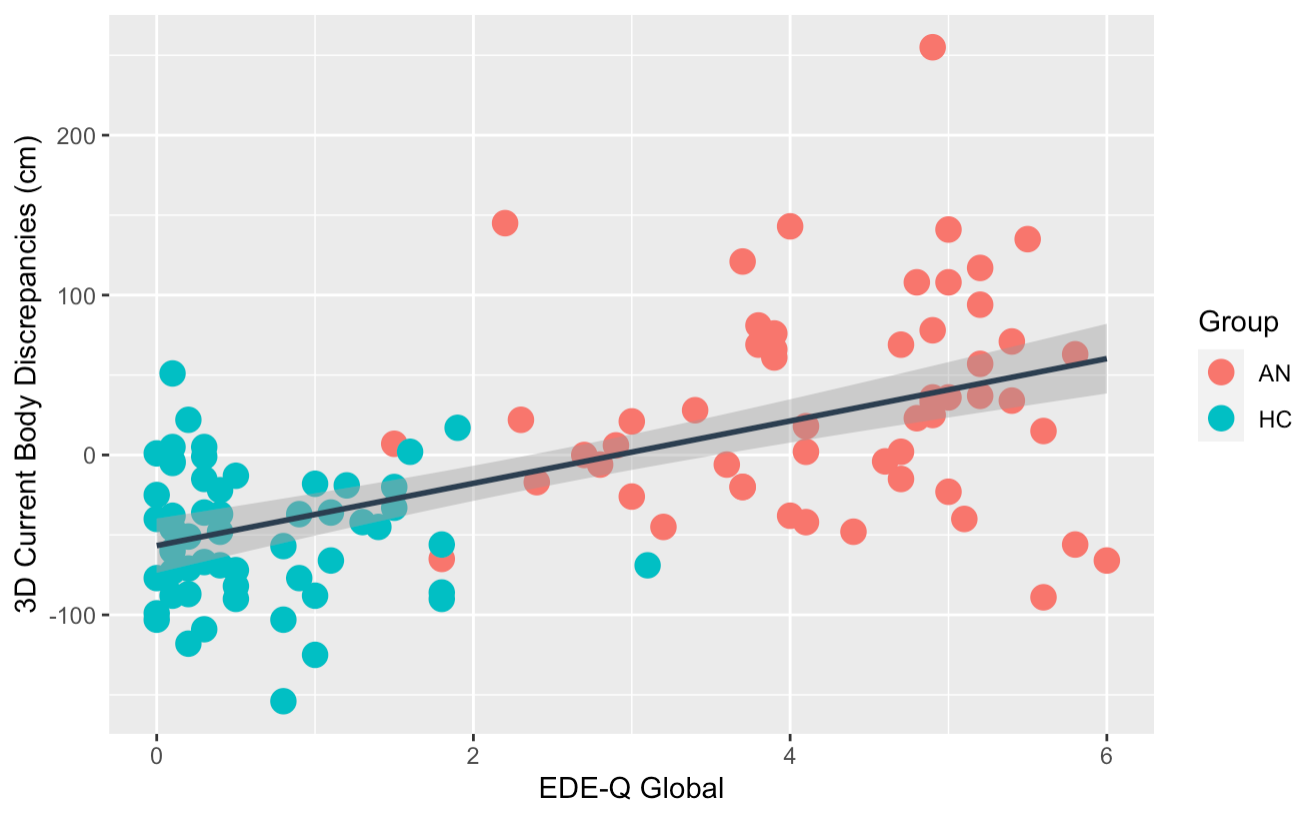
*

*R^2^* = .67; *F* (23,86) = 7.57, *P* < 0.001
